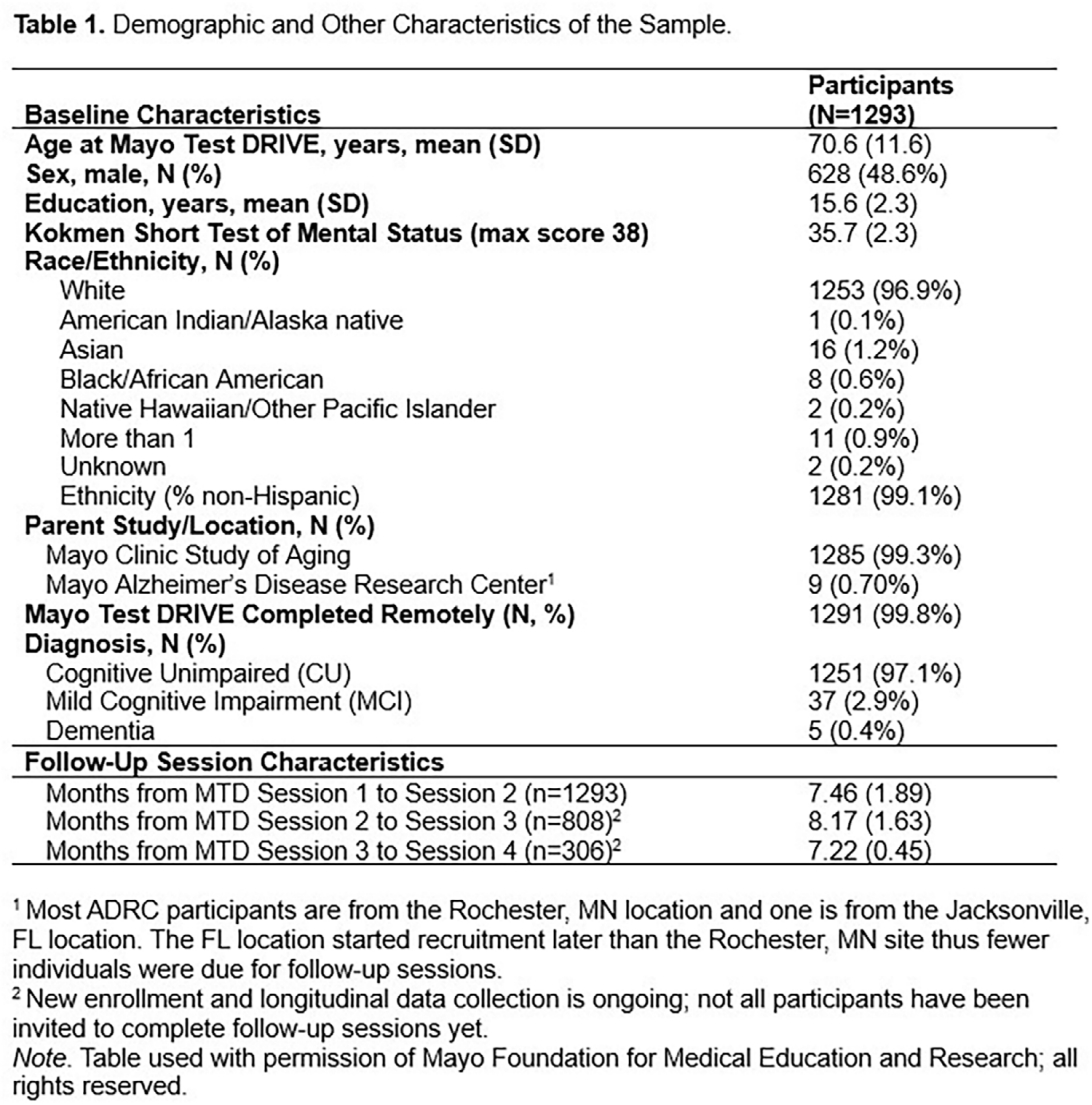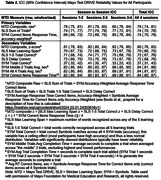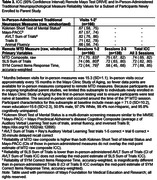# Reliability of the remote digital self‐administered Stricker Learning Span, Symbols Test and Mayo Test DRIVE Screening Battery Composite

**DOI:** 10.1002/alz.095558

**Published:** 2025-01-09

**Authors:** Morgan A Hughes, Ryan D. Frank, Winnie Z. Fan, Teresa J. Christianson, Walter K. Kremers, John L. Stricker, Elizabeth A. Boots, Mary M. Machulda, Julie A. Fields, Jason J. Hassenstab, Paula Aduen, John A. Lucas, Jonathan Graff‐Radford, Prashanthi Vemuri, Clifford R. Jack, David S. Knopman, Ronald C. Petersen, Nikki H. Stricker

**Affiliations:** ^1^ Mayo Clinic, Rochester, MN USA; ^2^ Washington University St. Louis, St. Louis, MO USA; ^3^ Mayo Clinic, Jacksonville, FL USA

## Abstract

**Background:**

Mayo Test DRIVE (MTD): Test **D**evelopment through **R**apid **I**teration, **V**alidation and **E**xpansion, is a web‐based multi‐device (smartphone, tablet, personal computer) platform optimized for remote self‐administered cognitive assessment that includes a computer‐adaptive word list memory test (Stricker Learning Span; SLS), a measure of processing speed/executive functioning (Symbols Test) and a score combining both the memory and processing speed/executive functioning measures (MTD Composite). We aimed to determine the reliability of MTD measures across follow‐up sessions.

**Method:**

Individuals participating in this ancillary study who completed at least two and up to four complete MTD sessions were included, resulting in 1,293 participants (mean age = 70.6, SD = 11.6; mean education = 15.6, SD = 2.3; 48.6% male; 96.9% White; 99.1% non‐Hispanic; 97.1% cognitively unimpaired) from parent studies (see Table 1). MTD raw scores were winsorized at 1% and 99% to minimize the impact of outliers. Test‐retest reliability was assessed using single‐rating, absolute‐agreement, and two‐way mixed ICCs and results are described using descriptives from Koo et al. (2016). ICCs for in‐person‐administered traditional neuropsychological measures are also reported alongside MTD for a subset of participants newly enrolled into the MCSA (to ensure participants were test naïve to both MTD and in‐person tests).

**Result:**

Reliability was good for the MTD Composite raw [total ICC = 0.78; see Table 2 for session‐to‐session ICCs and 95% CIs], and moderate‐to‐good for the primary outcome variables for each subtest [total ICC = 0.74 for both SLS and Symbols]. Weighting by accuracy negatively impacted reliability of Symbols and the Composite; without this weighting, MTD Composite and Symbols variables showed higher reliability (MTD Composite z total ICC = 0.80; Symbols response time total ICC = 0.84; see Table 2). Alternative methods of defining response time on Symbols showed nearly identical reliability values. SLS sum of trials (primary variable) had subtly higher reliability than separate measures of learning (1‐5 correct, max span) or delay. Reliability of remote MTD and in‐person‐administered measures and select comparisons are provided in Table 3.

**Conclusion:**

MTD showed moderate to good reliability, similar to in‐person‐administered traditional neuropsychological tests. Future work will examine the effect of demographics, device type, and session interference on reliability.